# Classification of Social Anxiety Disorder With Support Vector Machine Analysis Using Neural Correlates of Social Signals of Threat

**DOI:** 10.3389/fpsyt.2020.00144

**Published:** 2020-03-13

**Authors:** Mengqi Xing, Jacklynn M. Fitzgerald, Heide Klumpp

**Affiliations:** ^1^ Department of Psychiatry, University of Illinois at Chicago, Chicago, IL, United States; ^2^ Department of Psychology, Marquette University, Milwaukee, WI, United States; ^3^ Department of Psychology, University of Illinois at Chicago, Chicago, IL, United States

**Keywords:** social anxiety, neuroimaging, magnetic resonance imaging, machine learning, support vector machine

## Abstract

Threatening faces are potent cues in social anxiety disorder (SAD); therefore, neural response to threatening faces, particularly regions in the “fear” circuit such as amygdala, may classify individuals with SAD. Previous studies of indirect/implicit processing of threatening faces have shown that support vector machine (SVM) pattern recognition significantly differentiates individuals with SAD from healthy participants, though evidence for the role of the fear circuit in classification has been inconsistent. We extend this literature by using SVM during direct face processing. Individuals with SAD (n=47) and healthy controls (n=46) completed a validated emotional face matching task during functional MRI, which included a matching shapes control condition. SVM was based on brain response to threat (vs. happy) faces, threat faces (vs. shapes), and threat/happy faces (vs. shapes) in 90 regions encompassing frontal, limbic, parietal, temporal, and occipital systems. Recursive feature elimination (RFE) was used for feature selection and to rank the contribution of regions in predicting SAD diagnosis. SVM results for threat (vs. happy) faces revealed satisfactory accuracy (e.g., area under the curve=0.72); results with shapes as “baseline” yielded less optimal classification. RFE for threat (vs. happy) indicated that all 90 brain regions were necessary for classification. RFE-based ranking suggested diffuse neurofunctional activation to threat (vs. happy) faces in classification. When using an RFE cut-point, regions implicated in sensory and goal-directed processes contributed relatively more in differentiating SAD from controls than other regions. Results suggest that neural activity across large-scale systems, as opposed to fear circuitry alone, may aid in the diagnosis of SAD.

## Introduction

Social anxiety disorder (SAD) is one of the most common anxiety disorders in the United States (1) and a major public health problem. It is characterized by excessive fear and avoidance in a range of situations that involve potential negative judgment by others (2) and is associated with severe impairment (3–5). Yet, SAD is frequently underdiagnosed or misdiagnosed due in part to the shame and social evaluative fears intrinsic to the disorder (6, 7). Given that diagnostic accuracy is fundamental to appropriate treatment, an objective diagnostic approach has the potential to greatly reduce the burden and costs associated with SAD.

Extant functional magnetic resonance imaging (fMRI) studies have demonstrated aberrant activity in SAD relative to healthy controls (HC), suggesting that neurofunctional activity could serve as a classifier. In light of social fears, this work has generally used threatening facial expressions and focused on brain regions central to threat processing and the mediation of fear responses (e.g., amygdala, insula, infralimbic cortex) (8–10). While findings consistently point to exaggerated activity to threatening faces in the “fear circuit” in SAD relative to HC, atypical brain response (e.g., hyper- or hypoactivation) has also been observed in occipital, parietal, frontal, and subcortical regions in SAD [for reviews, see (11, 12)]. Findings suggest that disturbances in an array of regions implicated in emotion, sensory processes, and emotion regulation distinguish SAD from HC. However, neuroimaging results based on group effects (e.g., SAD vs. controls) do not delineate which regions predict SAD status at the individual level, the objective of brain-based markers of SAD.

The identification of neuromarkers for disease classification has both mechanistic and clinical utility. Subtle anomalies in the brain not detected with univariate model-driven methods may be captured with support vector machine (SVM) analysis, a data-driven, multivariate approach (13, 14). SVM employs a pattern recognition algorithm where a classifier trained on a subset of the data is used to predict the categories (e.g., patients or HC) according to new observations (i.e., “test” data).

SVM has demonstrated that neural activity during *indirect* face processing differentiates individuals with SAD from HC. A study by Frick and colleagues (15) consisted of 12 healthy male participants and 14 males with a primary diagnosis of SAD, without a comorbid depressive disorder. Whole-brain informed SVM results showed that activation when identifying the gender (male/female) of a threatening face significantly differentiated SAD from HC [area under the curve (AUC) = 0.70]. Additionally, SVM was performed with selected regions such as fear network (amygdala, anterior cingulate cortex, hippocampus, insula) and parietal lobe. Results showed that activation in the fear network also distinguished SAD from HC (AUC = 0.75), though its association with symptom severity is not clear as such data were not reported. In contrast to the fear network, parietal lobe responses were less effective in classifying SAD (AUC = 0.45) (15).

In a separate study, 16 individuals with SAD (16) and 16 individuals with panic disorder (without a comorbid depressive disorder) along with 19 HC completed an indirect face processing task where threatening and neutral faces were colored in red, yellow, or blue and participants were instructed to name the color of the face. For feature selection, leave-one-out cross-validation was used. Namely, one subject was withheld from the data set and a two-sample t-test was performed for the remaining training data. The features were ranked by absolute t-score, and the top number of features selected were used to predict the class of the withheld test data during the classification stage. Findings were not significant for face-color identification (AUC < 0.55). However, the feature selection approach ranking connectivity by the t-score of the two-sample t-test identified functional connectivity between the hippocampus and temporal pole and functional connectivity between middle temporal gyrus and frontal orbital cortex as key classifiers in distinguishing SAD participants from those with panic disorder (AUC = 0.81) and HC (AUC = 0.88) (16). Despite evidence that such functional connectivity served as classifiers, there were no associations with anxiety symptoms among participants with SAD.

Although inconsistencies were possibly due to methodological differences across studies including participant sample characteristics, findings suggest that neural activity during indirect/implicit face processing predicts SAD diagnosis. Also, despite small sample sizes, overall accuracy as represented by AUC ranged from 0.70 to 0.88, indicating adequate classification as 1 signifies perfect classification and 0.50 is no better than guessing (17). Altogether, SVM is a promising approach toward identifying brain-based biomarkers to detect SAD at the single-subject level.

The objective of the current study was to expand on the literature by conducting SVM classification with neural activity during *direct* threat face processing, an ecologically valid stimulus. Based on previous studies and contemporary models of SAD (11), we hypothesized that neural activity to threatening facial expressions in occipital, parietal, frontal, and subcortical regions would predict SAD diagnosis and that classification performance would be largely determined by regions (i.e., features) central to the fear circuit (e.g., amygdala, insula).

Although SVM is relatively insensitive to the size of features (18), overfitting commonly occurs in SVM and other machine learning models when the number of the features (e.g., brain regions) is similar or higher than the number of observations (e.g., participants). Thus, we used recursive feature elimination (RFE) in conjunction with SVM to test whether feature selection improved classification (19, 20). Lastly, we tested for potential relationships between brain regions that largely contributed to classification and symptom severity in the SAD group.

## Methods

### Participants

The study comprised 48 participants with SAD and 46 demographically matched HC, who met criteria for quality via visual inspection (e.g., integrity of coregistration) and quantitative parameters (e.g., movements were < 3 mm and < 3 degrees rotation in any one direction) during fMRI. However, one SAD participant was excluded due to a technical error during fMRI; thus, the SAD group comprised 47 participants. Exclusion criteria for all participants included: treatment (pharmacotherapy or psychotherapy), a comorbid depressive disorder, presence of a medical or neurological illness, less than 18 or more than 65 years of age, contraindications to magnetic resonance imaging (e.g., pregnancy, ferrous objects), current substance dependence (within 6 months of the study), or current or history of cognitive dysfunction (e.g., traumatic brain injury, pervasive developmental disorder). Additional exclusion criteria for HCs included a current or past Axis I disorder. A trained master's-level clinician performed the Structured Clinical Interview for DSM-5 (“SCID-5”) (21) and other clinician-administered measures. The clinician-administered Liebowitz Social Anxiety Scale (LSAS) (22) was used to assess social anxiety, and the Hamilton Anxiety Rating Scale (HAM-A) (23) and Hamilton Depression Rating Scale (HAM-D) (24) were used to evaluate anxiety and depression levels, respectively. All measures were collected within two weeks of the fMRI scan. All participants tested negative on a urine toxicology screen before the scan.

Procedures were approved by the local Institutional Review Board at the University of Illinois at Chicago and complied with the Helsinki Declaration, and participants were compensated for their time.

### fMRI Task

**Figure 1:**
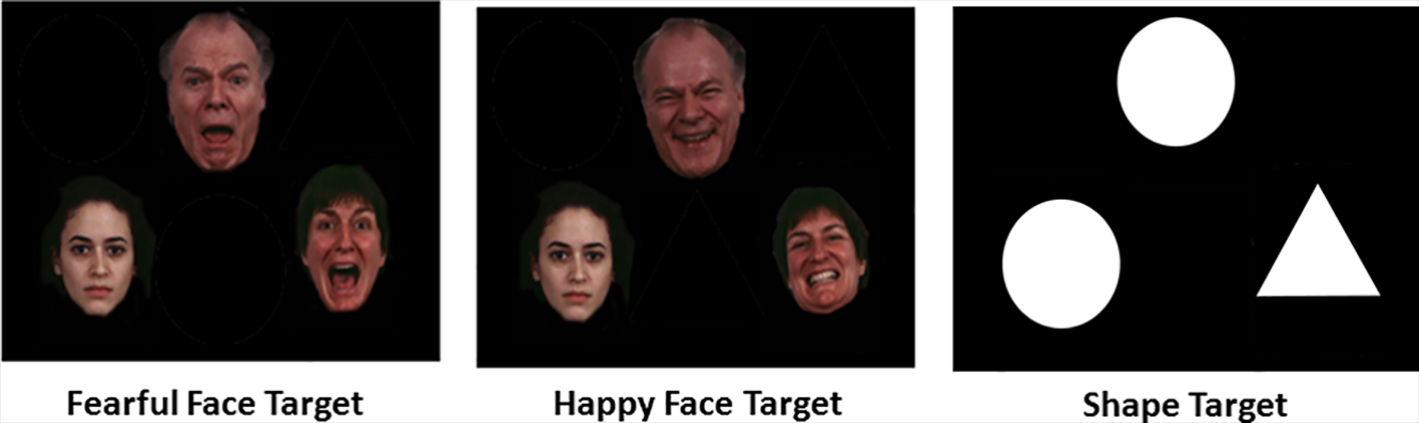
Schematic of Emotional Face Matching Task conditions (25).

During scanning, all participants performed a validated emotional face matching task (EFMT, see **Figure 1** for schematic), which has been used in studies involving SAD participants (26, 27) and is designed to probe brain response to signals of threat (i.e., angry, fearful faces) against those that do not convey threat (i.e., happy faces). The task comprised photographs from a validated set of face stimuli (25) presented in a block design, during which participants viewed a trio of faces (one target at top, two probes on bottom) and selected by right-handed button press which probe matched the target facial expression. The target and congruent probe face displayed one of three expressions (fearful, angry, or happy), whereas the incongruent probe face always displayed a neutral expression. Trials with emotional faces (fearful, angry, or happy) were interleaved with blocks of shapes (triangles, circles, squares) as a sensorimotor control condition, counterbalanced across a run. Three angry, three fearful, and three happy blocks of trials were interspersed with nine shape-matching blocks. Each block lasted 20 s and consisted of four back-to-back 5-s trials.

To maximize threat signal, angry and fearful faces were collapsed. While the primary contrast of interest was threat (vs. happy) faces, since happy faces as a “baseline” to threat has high ecological validity, classification was also performed for threat (vs. shapes) and all emotional faces (vs. shapes) to explore whether neural activity against a sensorimotor control condition improved classification.

Previous studies have shown that SAD and healthy participants are similar in task performance for the EFMT (26, 27). Nonetheless, to explore potential group differences, accuracy and reaction time for target threat (angry/fearful) and happy faces were submitted to a mixed 2 (Group: SAD, HC) x 2 (Emotion: threat, happy) analysis of variance (ANOVA) with repeated measures on the last factor.

To evaluate relationships between neural predictors and symptom severity, two-tailed Pearson's correlations were performed. All analyses were performed in the Statistical Package for the Social Sciences (SPSS, Version 24) unless otherwise stated.

### fMRI Data Acquisition and Preprocessing

Scanning was conducted on a 3 Tesla GE Discovery System (General Electric Healthcare; Waukesha, WI) with an 8-channel head coil. Functional data were acquired using gradient-echo echo planar imaging (EPI) sequence with the following parameters: TR = 2 s, TE = min Full [~25 ms], flip angle = 90°, FOV = 22 x 22 cm^2^, acquisition matrix 64 x 64, 3-mm slice thickness, 44 axial slices, 180 volumes per run. For anatomical localization, a high-resolution, T1-weighted volumetric anatomical scan was acquired.

The first four volumes from each run were discarded to allow for T1 equilibration effects. Conventional preprocessing steps were used in the Statistical Parametric Mapping (SPM8) software package (Wellcome Trust Centre for Neuroimaging, London www.fil.ion.ucl.ac.uk/spm). Briefly, images were temporally corrected to account for slice time acquisition differences and spatially realigned to the mean image to correct for head movement, while six motion parameters were entered as regressors of no-interest to control for minimal head movement during scanning. Images were then normalized to a Montreal Neurological Institute (MNI) template using the echo-planar imaging template, resampled to 2 x 2 x 2 voxels and smoothed with an 8 mm isotropic Gaussian kernel.

A general linear model was applied to the time series, convolved with the canonical hemodynamic response function and with a 128s high-pass filter. Using a box-car model, the contrasts of interest—threat (vs. happy) faces, threat (vs. shapes), and all faces (vs. shapes)—were generated for each participant.

### Classification

The Automated Anatomical Labelling atlas with MarsBar for SPM8 (28, 29) was used to generate regions of interest (ROIs) within the frontal, parietal, temporal, and occipital cortices, and subcortical system, which totaled 90 regions. An SVM classification model that was constructed with brain activation [β weights, arbitrary units (a.u.)] derived from each of these anatomy-based ROIs was in Python 3.6 [Guido van Rossum, Centrum Wiskunde & Informatica (CWI), Netherlands]. Linear kernel was selected to define the support vector (i.e., hyperplane) with default settings in scikit-learn (30). Classifier performance was examined with AUC, sensitivity, and specificity.

Feature selection was performed with RFE. If RFE eliminated brain regions, a nonparametric permutation test was used to test whether AUC between the original SVM model and SVM+RFE model significantly differed from each other. Regardless as to whether RFE eliminated brain regions or not, RFE yields a Fisher score (31) to rank the importance of the feature (i.e., brain region). Therefore, the Fisher score was used to examine the contribution of brain regions in classification. To test the generalizability for classification results, 10-fold cross-validation (leave one out) was performed.

## Results

### Participants

Two-tailed independent *t*-tests and chi-square tests were performed to evaluate participant characteristics. Results showed that the SAD group was more socially anxious [LSAS; *t*(91) = 24.01, *p *< 0.001], generally anxious [HAM-A; *t*(91) = 11.21, *p*<0.001], and depressed [HAM-D; *t*(91) = 0.99, *p *< 0.001] than the HC group. However, groups were similar in age [*t*(91)=0.03, *p *= 0.97] and education in years [*t*(91) = 0.27, *p *= 0.79]. The distribution of gender *χ^2^* = 0.12, *p* = 0.81 and race/ethnicity *χ^2 ^*= 3.45 *p *= 0.75 were also comparable between groups. See **Table 1** for demographics and clinical details.

**Table 1:** Demographic and clinical characteristics and task performance.

	Social Anxiety Disorder (N=47)		Heathy Controls (N=46)	
	M(SD)		M(SD)	
LSAS	79.31(15.0)		14.0(10.8)^a^	
HAM-A	12.0 (6.5)		0.9 (1.5)^a^	
HAM-D	6.7 (3.9)		0.6 (1.1)^a^	
Age	25.7 (6.2)		25.8 (8.4)	
Education in years	15.7 (2.0)		15.6 (2.4)	
**Race/Ethnicity**	**N**	**%**	**N**	**%**
Caucasian	31	66.0	24	52.2
Asian	9	19.1	13	28.3
African American	3	6.4	4	8.7
American Indian or Alaskan Native	1	2.1	1	2.2
More than one race or unknown	3	6.4	4	8.7
Hispanic	10	20.8	11	23.9
**Gender**	**N**	**%**	**N**	**%**
Male	13	27.7	12	26.1
Female	34	72.3	34	73.9
**Comorbidity**	**N**	**%**	–	
Generalized anxiety disorder	15	31.9	–	
Persistent depressive disorder	6	12.7	–	
Specific phobia	5	10.6	–	
Panic disorder	4	8.5	–	
Posttraumatic stress disorder	2	4.3	–	
**Task Performance**	**M(SD)**		**M(SD)**	
Response time for threat faces (milliseconds)	1348.3 (398.5)		1416.3 (349.7)	
Response time for happy faces (milliseconds)	1324.3 (299.0)		1426.4 (347.7)	
Accuracy for threat faces (%)	89.8 (8.8)		90.5 (9.5)	
Accuracy for happy faces (%)	96.3 (10.4)		97.1 (7.8)	

LSAS, Liebowitz Social Anxiety Scale; HAM-A, Hamilton Anxiety Rating Scale; HAM-D, Hamilton Depression Rating Scale

a Healthy controls were less socially anxious (LSAS), less generally anxious (HAM-A), and less generally depressed (HAM-D) than participants with social anxiety disorder (p<0.05).

### Behavioral Results

The ANOVA for accuracy revealed a main effect of Emotion [*F*(1, 91) = 75.06, *p*<0.001] but no main effect of Group [*F*(1, 91) = 0.00, *p *= 0.99] or Emotion x Group interaction [*F*(1, 91)=0.01, *p *= 0.94]. Follow-up paired t-tests showed that accuracy was lower when identifying target threatening faces than happy faces [*t*(92) = 8.71, *p*<0.005]. The same analysis for reaction time for accurate trials did not yield a main effect of Emotion [*F*(1, 91) = 0.27, *p *= 0.61], Group [*F*(1, 91) = 2.63, *p *= 0.11], or Emotion x Group interaction [*F*(1, 91) = 0.05, *p *= 0.83]. Details are reported in **Table 1**.

### Feature Selection and Support Vector Machine Performance

**Figure 2:**
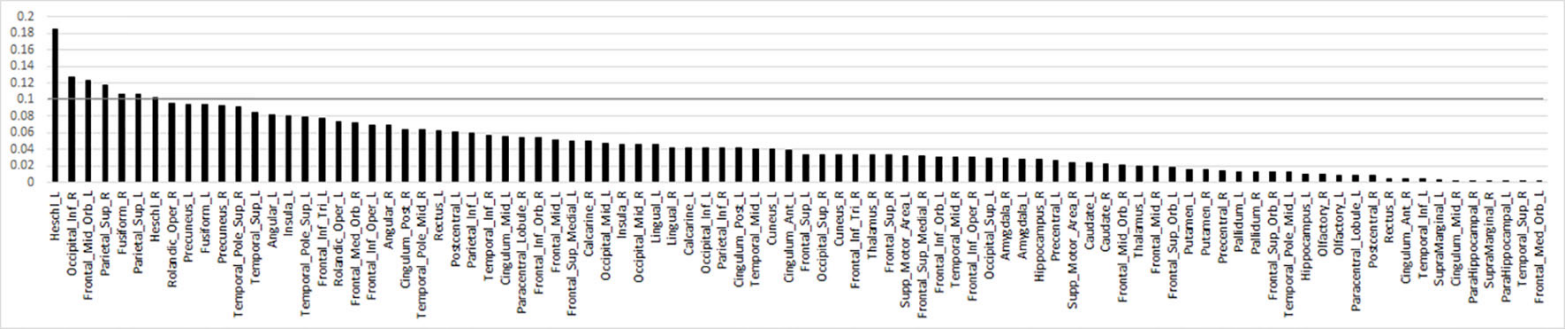
Bar plot of the Fisher score ranks for anatomy-based regions of interest related to brain response to threat (>happy) faces in descending order. Line denotes arbitrary Fisher score cut-point of 0.10 to highlight regions that largely differentiated individuals with social anxiety disorder from healthy controls.

For the primary contrast of interest, threat (vs. happy) faces, no regions were excluded based on RFE. Results for the original SVM model with 90 ROIs achieved a cross-validated accuracy of 69.98%, with AUC = 0.72 (*p*<0.001); sensitivity = 0.71 and specificity = 0.69. RFE feature importance (i.e., Fischer score) results yielded a smooth decay across ROIs as opposed to a robust inflection separating high and low feature importance (**Figure 2**). Due to the smooth decay, we used an arbitrary cut-point of 0.10 based on the Fisher score to evaluate relationships between brain regions (i.e., features) that contributed relatively more to classification and symptom severity. Regions with a relatively high Fisher score (i.e., >0.10) were bilateral Heschl's gyrus, right inferior occipital gyrus, left middle orbitofrontal gyrus, bilateral superior parietal gyrus, and right fusiform gyrus. Pearson's correlations within the SAD group did not reveal significant relationships between activity in these regions and social anxiety severity (LSAS) or general anxiety level (HAM-A) (all *p's* > 0.05).

Regarding threat faces (vs. shapes), RFE excluded 73 regions; for all faces (vs. shapes), RFE excluded 29 regions. Yet, when classification was performed with SVM+RFE or SVM alone (i.e., all 90 regions), accuracy was not as high or sensitivity/specificity as balanced as for threat (vs. happy) faces for either threat faces (vs. shapes) or all faces (vs. shapes). See **Supplementary Table 1** for details.

## Discussion

This is the first study we are aware of that used SVM to classify individuals with SAD based on brain response during direct threat processing with a validated emotion processing task. With regard to behavioral performance, accuracy was high, verifying that all participants followed task instructions. SAD was predicted by widespread activity to threat (vs. happy faces), and classification accuracy (i.e., AUC), sensitivity, and specificity were satisfactory and consistent with previous neuroimaging studies that used SVM to predict SAD in the context of indirect/implicit face processing (15, 16) in addition to resting state (32).

**Figure 3:**
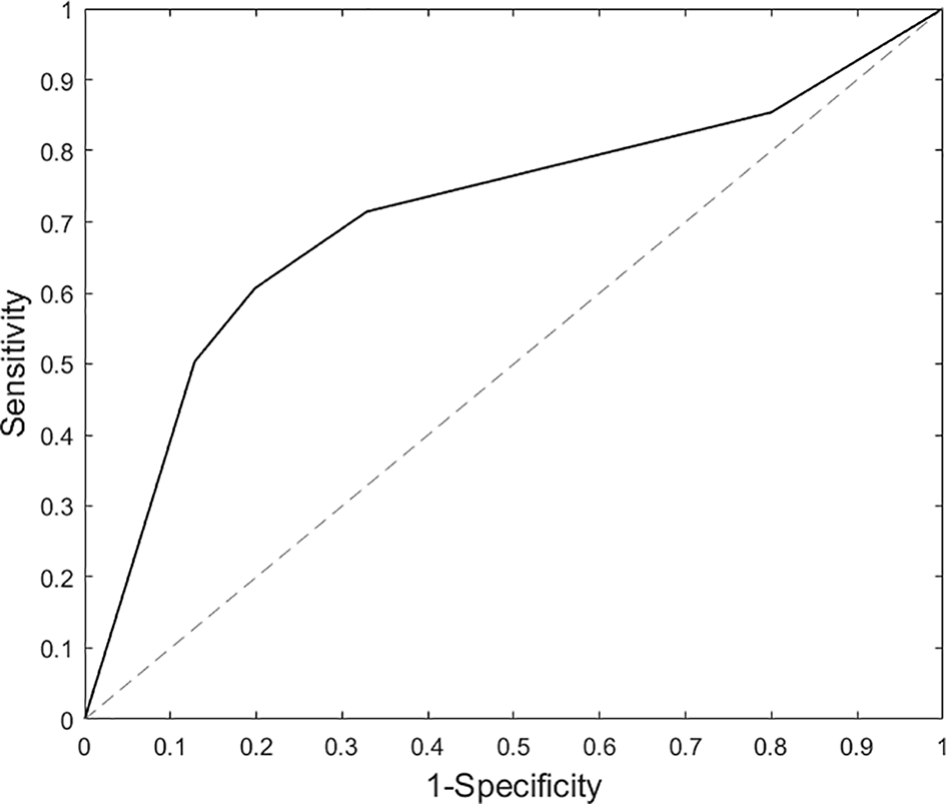
Area under the curve results based on support vector machine analysis for threat (vs. happy) faces.

Findings partially support our hypotheses as brain response to threatening (vs. happy) faces classified SAD participants; however, feature selection did not improve classification as neural activity in all ROIs was required to distinguish SAD participants from HC (i.e., AUC = 0.72, **Figure 3**), with cross-validation accuracy ~70% and specificity and sensitivity ~0.70%. Findings suggest that neural activation differences between SAD and HC are diffuse; thus, an array of brain regions supporting various functions were needed to identify the underlying patterns for a binary classifier. Results are similar to Frick and colleagues (15) insofar as they also showed that whole brain activity significantly differentiated SAD participants from HC.

Evidence that neural activity across occipital, parietal, frontal, and subcortical regions predicted SAD diagnosis supports our hypothesis. However, we also expected regions (i.e. features) that underlie that the fear circuit (e.g., amygdala, insula) would largely contribute to classification that was not observed. Though these regions were needed to obtain satisfactory classification, their Fisher scores were relatively low, indicating that they made a necessary but not substantial contribution. It is possible that SAD patients had similar amygdala hyperreactivity to both threat and happy faces, as an earlier study reported exaggerated amygdala reactivity to threat and happy faces in SAD relative to controls (33). Individual differences in amygdala response to emotional faces in SAD may have reduced the ability for amygdala to robustly predict diagnostic status.

When using an arbitrary cut-point based on Fisher scores generated by RFE feature selection, regions that “largely” contributed to diagnostic classification (i.e., had relatively high Fisher scores) comprised structures involved in sensory functions. Inferior occipital gyrus and fusiform gyrus support higher-order visual processing and face perception (34), parietal superior gyrus responds to visual input and is involved in spatial orientation (35), middle orbitofrontal gyrus has extensive connections with sensory and limbic structures (36) and is implicated in goal-directed attention (e.g., selection of task-relevant stimuli and responses; superior parietal cortex, frontal middle gyrus) (37, 38), and Heschl's gyrus engages to auditory stimuli (39).

Cognitive models propose that biases to negatively valenced stimuli play a critical role in anxiety by forming, or reinforcing, maladaptive views of the self and others (40, 41). In support, behavioral studies consistently demonstrate attentional bias to threat faces in SAD (42–44) and preferential attention to threat is thought to contribute, in part, to the development and maintenance of SAD (45, 46). Consequently, occipital, fusiform, parietal superior, and middle orbitofrontal gyrus activation may factor into predicting SAD diagnosis given the salience of threatening faces when contrasted with a positive/approach socioemotional signal. We speculate that the background scanner noise may explain the Heschl's gyrus finding as such noise has been shown to influence activity in this region in healthy participants (47). For example, SAD patients report poorer attentional control than healthy individuals (48) and thus may be more susceptible to being distracted by vibrations of the gradient coil or other features of the MRI environment. Since our study was not designed to test this, further investigation is needed to understand the role of Heschl's gyrus in predicting SAD diagnosis.

Despite the contribution of these regions in classification, there was no association between neural activity and symptom severity, which is consistent with a prior SVM SAD study that performed correlational analysis (16). The lack of a robust inflection separating high and low Fisher scores may have reduced our ability to detect significant associations. Even so, links between illness severity and neurofunctional activity in SAD may be tenuous. For example, associations between amygdala activity, a region implicated in the neurobiology of SAD (11, 12), and social anxiety severity have been inconsistent (49–51). It is possible that variance in neurofunctional activity may not be strongly tied to psychological measures, which are relatively distal measures of biology and subject to inaccuracy (e.g., negative bias) (52).

In addition to our primary contrast of interest (i.e., threat vs. happy), we explored classification based on neural activity to threat faces (vs. shapes) and emotional faces (vs. shapes). Interestingly, RFE did exclude certain brain regions to improve classification, yet, accuracy for SVM+RFE and SVM alone tended to be lower and there was more imbalance between sensitivity and specificity. Using a sensorimotor control condition as a baseline may have introduced more noise and complexity in classification than a facial expression as baseline.

Altogether, differential neural activity between threatening and positive socioemotional cues may serve as a biomarker to detect SAD at the single-subject level. However, it will be important for future studies to test the extent to which this may (or may not) generalize to other internalizing psychopathologies (e.g., depression, generalized anxiety disorder). Indeed, contemporary models of psychopathology emphasize a transdiagnostic, dimensional approach based on observations that behavioral and neurobiological substrates cut across standard diagnostic categories (53, 54). Accordingly, it may be more impactful to identify biomarkers that map onto constructs shared across disorders, for example, attentional control or executive function, which modulate emotional reactivity (55, 56) and are disrupted to varying degrees in individuals with psychiatric illness (57, 58).

Findings should be considered in light of limitations. Certain comorbidity was permitted; therefore, results may not extend to the classification of SAD alone. In addition, we used an AAL-based atlas in the creation of brain ROIs. Alternative approaches to ROI creation exist (59, 60); consequently, differences in the selection of regions to include in the SVM analysis may have impacted results. Also, findings may not generalize to indirect/implicit face processing or to other stimuli (e.g., images of general negative content, salient words). Further, to standardize the analyses, the classification adopted the default parameter with a linear kernel provided by the toolbox, which may affect the ability in recognizing complex patterns during the feature selection. More complex kernels and parameters can be explored in the future application to potentially enhance the performance during the feature selection. Lastly, we used cross-validation to examine the generalizability of classification performance; however, it will be important to replicate results in an independent sample before drawing firm conclusions.

## Conclusions

Despite limitations, the current study provides evidence that SVM based on brain response to threat faces is a promising approach for classifying SAD. Whole-brain activity to threat (vs. happy) faces was required for optimal classification performance. Brain regions showing relatively higher importance in classification highlight the relevance of brain regions outside the fear circuit (e.g., amygdala, insula) in predicting SAD. In particular, results suggest that activity in regions that govern sensory and goal-directed processes may play a role in SAD diagnosis.

## Data Availability Statement

Requests to access the data sets will be considered upon request to the corresponding author. However, data sets may not be complete as not all participants may have provided consent to share their raw data.

## Ethics Statement

The studies involving human participants were reviewed and approved by Office for the Protection of Research Subjects, University of Illinois at Chicago. The patients/participants provided their written informed consent to participate in this study.

## Author Contributions

MX contributed to the analysis and interpretation of results. JF contributed to manuscript preparation and interpretation of results. HK contributed to the research design, manuscript preparation, and interpretation of results.

## Funding

This work was supported by NIH/NIMH K23MH093679, R01MH112705, and in part by R01MH101497, and the Center for Clinical and Translational Research (CCTS) UL1RR029879. JF was supported by F32MH117895.

## Conflict of Interest

The authors declare that the research was conducted in the absence of any commercial or financial relationships that could be construed as a potential conflict of interest.
