# Supplementary material for: Classification of Social Anxiety Disorder With Support Vector Machine Analysis Using Neural Correlates of Social Signals of Threat
Source: Front Psychiatry. 2020 Mar 13;11:144. doi: 10.3389/fpsyt.2020.00144 (PMC7082922; doi:10.3389/fpsyt.2020.00144)
Supplement: Supplementary file 1 [file Table_1.docx]

**Supplementary Table 1: Support Vector Machine Results with Shapes as Baseline**

| **Task Condition** | **10 folds Cross-Validation**  **Accuracy** | **Number of ROIs included** | **Area Under the Curve** | **Sensitivity** | **Specificity** |
| --- | --- | --- | --- | --- | --- |
| Threat vs. Shapes (SVM) | 54.5% | 90 | 0.40 | 0.84 | 0.27 |
| Threat vs. Shapes  (SVM+RFE) | 67.5% | 17 | 0.61 | 0.87 | 0.41 |
| Faces vs. Shapes (SVM) | 59.6 % | 90 | 0.53 | 0.92 | 0.22 |
| Faces vs. Shapes  SVM+RFE) | 63.3% | 61 | 0.54 | 0.83 | 0.39 |

ROIs=Regions of Interest; SVM=Support Vector Machine; RFE=Recursive feature elimination
